# Profiling of metalloprotease activities in cerebrospinal fluids of patients with neoplastic meningitis

**DOI:** 10.1186/s12987-017-0070-5

**Published:** 2017-08-14

**Authors:** Catharina Conrad, Kristina Dorzweiler, Miles A. Miller, Douglas A. Lauffenburger, Herwig Strik, Jörg W. Bartsch

**Affiliations:** 1 Department of Neurology, Philipps University Marburg, Baldingerstr, 35033 Marburg, Germany; 2 Department of Anesthesiology and Intensive Care Medicine, University Hospital, Albert-Schweitzer Campus 1, 48149 Münster, Germany; 3 Department of Neurosurgery, Philipps University Marburg, Baldingerstr, 35033 Marburg, Germany; 4 Department of Biological Engineering, Massachusetts Institute of Technology, Cambridge, MA 02139 USA; 5 Center for Systems Biology, Massachusetts General Hospital, Harvard Medical School, Boston, MA 02114 USA

**Keywords:** Neoplastic meningitis, Metalloproteases, CSF, Real-time protease activities, FRET-substrates

## Abstract

**Background:**

Neoplastic invasion into leptomeninges and subarachnoid space, resulting in neoplastic meningitis (NM) is a fatal complication of advanced solid and hematological neoplasms. Identification of malignant involvement of the cerebrospinal fluid (CSF) early in the disease course has crucial prognostic and therapeutic implications, but remains challenging. As indicators of extracellular matrix (ECM) degradation and breakdown of the blood–brain-barrier, Matrix Metalloproteases (MMPs) and A Disintegrin and Metalloproteases (ADAMs) are potential analytes for cerebral pathophysiology and metastatic dissemination of tumor cells into the CSF.

**Methods:**

We compared protease activities in CSF samples from patients with NM and control individuals using FRET-based metalloprotease substrates with distinct enzyme selectivity profiles in a real-time, multiplex approach termed “proteolytic activity matrix assay” (PrAMA). Protease activity dynamics can be tracked by fluorescence changes over time. By simultaneously monitoring a panel of 5 FRET-substrate cleavages, a proteolytic signature can be identified and analyzed to infer the activities of multiple specific proteases. Distinct patterns of substrate cleavage comparing disease vs. control samples allow rapid, reproducible and sensitive discrimination even in small volumes of CSF.

**Results:**

Individual substrate cleavage rates were linked to distinct proteases, and PrAMA computational inference implied increased activities of MMP-9, ADAM8 and ADAM17 (4–5-fold on average) in CSF samples from NM patients that were inhibitable by the metalloprotease inhibitor batimastat (BB-94). The activities of these proteases correlated with blood–brain barrier impairment. Notably, CSF cell counts were not found to directly reflect the protease activities observed in CSF samples from NM patients; this may explain the frequent clinical observation of negative cytology in NM patients.

**Conclusion:**

PrAMA analysis of CSF samples is a potential diagnostic method for sensitive detection of NM and may be suitable for the clinical routine.

**Electronic supplementary material:**

The online version of this article (doi:10.1186/s12987-017-0070-5) contains supplementary material, which is available to authorized users.

## Background

Leptomeningeal metastasis (LM) resulting in Neoplastic Meningitis (NM) is a spread of malignant cells to the leptomeninges, the subarachnoid space and dissemination of tumor cells within the cerebrospinal fluid (CSF). NM is a complication of patients with progressive cancer (70%) and prognosis is poor once manifest NM with neurological deficits develops [1]. Patients with breast cancer, lung cancer, malignant melanoma and hematopoietic neoplasms are affected most frequently [1, 2].

Recently, the combination of neurological examination, radiographic imaging of the neuroaxis and CSF cytology are used to diagnose NM [3, 4]. However, all these techniques suffer from limitations and may only detect NM at an advanced stage of malignant CSF invasion. CSF cytology has the highest specificity in diagnosis and detects tumor cells in the CSF in >95%, however the sensitivity of this method is generally less than 50% [3]. Depending on the quality of the cytospin preparation and the expertise of the clinical professional, the diagnostic accuracy of the CSF analysis is variable [2, 4]. Early diagnosis of NM as well as therapy monitoring is crucial for the patient outcome and an important prerequisite for disease control, since NM of solid tumors without therapy progresses to death within 4–6 weeks accompanied by severe neurological symptoms [5, 6]. To overcome the lack of sensitivity, research has focused on biomarkers and characterization of tumor cells in the CSF [7–10]. Proteomic approaches attempted to relate differential CSF protein profiles obtained from mass spectrometry-based studies to neoplastic diseases to identify potential biomarkers [11, 12]. Nevertheless, it is difficult to attribute disease identification and progression to a single protein. CSF profiling of the biological activity of multiple biomarkers may more accurately reflect the pathophysiological processes than one biomarker, increasing specificity and sensitivity [13].

The metastatic invasion of tumor cells and dissemination to secondary sites is promoted by deregulated activity of extracellular metalloproteases (MPs), such as matrix metalloproteases (MMPs) and A Disintegrin and Metalloproteases (ADAMs). Both classes of proteases are members the metzincin superfamily, meaning that they utilize a complexed zinc ion in their catalytic site to constitute proteolytic activity. Major roles for MMPs and ADAMs in cancer progression have been reported, in particular, tumor cell invasion, angiogenesis and metastasis [14, 15]. Cancer promoting protease function is part of a tightly regulated multidirectional network of numerous proteolytic enzymes and their physiological inhibitors, which is modulated context-dependent and involves proteolytic interactions of tumor, stromal and infiltrating immune cells [16]. The biological activities of MMPs and ADAMs in the context of neoplastic meningitis remain less clear but likely given the dissemination of tumor cells through the Virchow–Robin space into the CSF as outlined (Additional file 1: Fig. S1). The occurrence of extracellular proteases in the CSF has been reported under various pathological conditions of the brain including infection [17–21], inflammation [22–26], neurodegeneration [27–32], ischemia [33, 34] and neoplasm [35, 36]. Proteolytic enzymes involved in the disturbance of tissue integrity and the penetration of tumor cells into the subarachnoid space could serve as diagnostic markers for NM [37, 38]. Thus, the analysis of specific patterns of protease activation in the CSF may reflect whether a condition is benign or malignant, and may be a suitable approach for specific and sensitive detection of NM.

In order to obtain metalloprotease profiles for patients with NM in a pilot study, we utilized a multiplex approach for simultaneous detection of MMP and ADAM activities in the CSF of patients with NM compared to control individuals and tumor patients without NM. The Proteolytic Activity Matrix Analysis (PrAMA) is a combined experimental and mathematical method based on time-lapse fluorescence measurements of a panel of moderately specific FRET-based polypeptides in small volumes of biological samples. The observed cleavage patterns are compared to a standard table of catalytic efficiencies measured from purified mixtures of recombinant metalloproteinases and should reflect changes in specific enzyme activities [13, 39] in CSF samples from patients affected by NM.

## Methods

### Recruitment of patient cohorts and collection of cerebrospinal fluid (CSF)

This pilot study obtained ethical approval from the local Ethics Committee (Marburg University; File No. 101/15). Informed consent was provided from patients to use their biological specimens and clinicopathological data for research purposes. No selection criteria were applied, i.e. all available patients with suspected diagnosis of NM and with brain metastases undergoing routine lumbar puncture were recruited into the study. CSF samples in the control group were collected during therapeutic lumbar punctures from patients with normal pressure hydrocephalus (3/12) or idiopathic intracranial hypertension (9/12). Patients with normal CSF cytology and blood–brain-barrier (BBB) physiology without clinical evidence for neuro-infectious, neuro-inflammatory, neuro-degenerative or neoplastic brain pathologies were considered as controls. All CSF samples were collected at the Department of Neurology, Philipps University Marburg, Germany. Lumbar puncture to collect CSF was performed by medical staff according to clinical guidelines. Upon collection, CSF served primarily for diagnostic purposes, remaining CSF was placed on ice and centrifuged at 1000*g* to remove cells. Clarified CSF was aliquoted, snap-frozen in liquid nitrogen and stored at −80 °C for further analysis. Clinicopathological features were documented pseudonymized, patients were grouped in control individuals (crtl), patients with neoplastic meningitis (NM) and patients with brain metastases without neoplastic meningitis (w/o NM) according to neurological examination, contrast-enhanced MRI of the brain and neuroaxis and CSF cytology for clinical diagnosis.

### Determination of MMP/ADAM activities in CSF

Cell-free cerebrospinal fluids were tested for MMP/ADAM activity by using the Proteolytic Activity Matrix Analysis (PrAMA) technique developed by Miller et al. using FRET-based polypeptide substrates PEPDab005, PEPDab010, PEPDab008, PEPDab013 and PEPDab014 (BioZyme Inc, Apex, NC), which vary in their specificities towards different ADAM family members and MMPs. PrAMA analysis was performed as described earlier [13, 39]. Briefly, for time-lapse fluorimetry, a final substrate concentration of 10 μM (diluted from 5 mM stock in DMSO) in 50 μl of activity buffer (1 μM ZnCl_2_, 20 mM Tris–HCl pH 8.0, 10 mM CaCl_2_, 150 mM NaCl, 6 × 10^−4^% Brij-35) was incubated with 50 μl of CSF using 96-well microtiter white opaque plates, each sample was run in technical triplicates. Samples containing sufficient volumes were included for inhibitor studies and repetitive measurements. To some samples, the broad-range MMP/ADAM inhibitor batimastat (Tocris Bioscience, Bio-Techne, Wiesbaden, Germany) was added at a concentration of 1 μM dissolved in DMSO. Fluorescence units versus time were monitored with a Fluostar BMG Optima using excitation and emission wavelengths of 485 and 530 nM at 37 °C, respectively. A non-linear model was used for curve fitting as described previously [13], the signal of a negative control was subtracted (FRET-substrate only) and time-lapse fluorimetry data were normalized to a positive control (0.01% Trypsin). Specific protease activities were inferred with PrAMA by comparing the pattern of substrate cleavage rates for each sample to a matrix of known substrate specificities for ADAM8, ADAM17, MMP-2 and MMP-9 that was determined using purified enzymes [13]. All calculations and statistical evaluation of data was conducted using Matlab (2014b, MathWorks, Natick, MA).

### Statistical analysis

The increase in fluorescence resulting from substrate proteolysis was tracked every 5 min for 4 h. For interpretation of time-lapse fluorimetry data, a non-linear curve-fitting model that accounted for substrate depletion and photobleaching decay served to determine cleavage rates. Cleavage rates are all presented in heat maps averaged over technical triplicates, clear outliers were excluded using Dixon’s Q-Test with a 90% threshold. PrAMA inference was performed as described previously with 30% sampling error and threshold σ_T_ = 1.4 [13]. Based on normal distribution of values as tested by the David test at the significance level p = 90%, statistical significance was determined using a two-tailed unpaired Student’s *t test* to compare two sample groups. To compare more than two experimental groups, Analysis of Variance (ANOVA) was used. Values are denoted as not significant (ns, p ≥ 0.05), significant * (p ≤ 0.05), highly significant ** (p ≤ 0.01), or very highly significant *** (p ≤ 0.001).

To group the generated datasets, observed average cleavage rates were hierarchically biclustered mean-centered and variance-normalized by row, using Euclidean distance, average linkage and optimal leaf order. Clear patterns emerging from cluster analysis are indicated by dendrograms flanking the array.

To investigate the relationship between CSF cell count, blood–brain-barrier impairment and protease activities, Pearson’s correlation coefficients (r) were calculated between these parameters, respectively. Statistical significance (p) was determined as described in the section above.

## Results

### Patient groups

According to the clinical diagnosis of medical professionals at the department of Neurology, patients were assigned to one of three groups: patients with neoplastic meningitis (NM, n = 12), patients with brain metastases but without leptomeningeal tumor spread (w/o NM, n = 12) or controls (ctrl, n = 12). CSF controls were characterized by normal CSF cytology, physiological protein concentrations, normal albumin and immunoglobulin quotients and absence of oligoclonal IgG bands, thus excluding inflammation, infection and impairment of the blood–brain-barrier. Comparison of baseline demographics for each group did not show significant differences. The mean age was 58.0 years (range 48–60 years) for patients with NM, 53.5 years (range 24–75 years) for patients with brain metastases w/o NM and 41.2 years (range 20–68 years) for controls. In our patient cohort, the NM cases were solely from female patients (gender male/female: NM 0/12) since NM is most prevalent in breast cancer patients. Accordingly, we recruited patients w/o NM and control group as far as possible from other female patients so that the proportion of female patients was higher than males (gender male/female: w/o NM 3/9 and crtl 1/11).

### Protease activity is increased in the CSF of patients with neoplastic meningitis

**Fig. 1:**
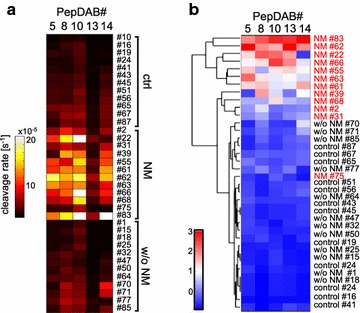
Analysis of protease activities in the CSF of three patient cohorts. Cleavage signatures in CSF samples were analyzed from 12 control individuals (crtl), 12 patients with neoplastic meningitis (NM) and 12 patients with brain metastases of different primary tumors, but without neoplastic meningitis (w/o NM). Protease activities in real-time mode were determined using PEPDab substrates 5, 8, 10, 13 and 14 at final concentrations of 10 µM for 4 h. Heat map summarizing mean cleavage rates of 3 technical replicates for each clinical samples calculated from time-lapse fluorimetry **a**. Hierarchical bi-clustering result corresponding to (**a**), with data mean-centered and variance-normalized by row. CSF cleavage patterns of patients with NM (labeled *red*) cluster closely together, control individuals and patients with brain metastases w/o NM group together without forming main clusters for each condition (**b**)

Clinically obtained CSF samples from controls, patients with NM and patients with brain metastases but no leptomeningeal metastasis were subjected to time-lapse fluorimetry and analyzed for substrate cleavage rates of FRET peptides PEPDab 5, 8, 10, 13 and 14 in parallel (Fig. 1a; Additional file 1: Table S1). Distinct patterns of protease activity for disease and control samples were found. Overall, the observed reaction rates showed the strongest activity with PEPDab#10, which is most efficiently cleaved by ADAM17 [13]. Second strongest substrate cleavage was detected for PEPDab#8, which is most selective for MMP-9. We discovered that enzymatic activity tracked by time-lapse fluorimetry increased in the CSF of patients with NM, whereas cleavage rates in the CSF of control individuals and patients with brain metastases w/o NM were similar and remained close to background fluorescence. Cleavage rates in the CSF were significantly different between ctrl and NM, as well as between NM and w/o NM for all PEPDab substrates analysed (p = 0.01 for PEPDab 5; p = 4.2 × 10^−6^ for PEPDab 8; p = 0.00013 for PEPDab 10; p = 0.09 for PEPDab 13; p = 0.0009 for PEPDab 14) whereas there were no significant changes detectable between crtl and patients with brain metastatic cancer w/o NM.

Resulting sample cleavage patterns were hierarchically bi-clustered, mean-centered and variance-standardized using Euclidean distance, average linkage and optimal leaf ordering (Fig. 1b). This type of cluster analysis groups 88% of the NM CSF samples in two main clusters as indicated by the dendrogram flanking the array; in contrast, substrate cleavage patterns in CSF samples of control individuals and patients with brain metastases w/o NM cluster closely together with no clear pattern. Of the NM CSF samples, sample#75 is the only one that does integrate into the group of neoplastic meningitis. These results suggest that disease samples can be clearly distinguished from control samples by their substrate cleavage signature.

### Inference of protease identities in CSF samples revealed by the PrAMA method

**Fig. 2:**
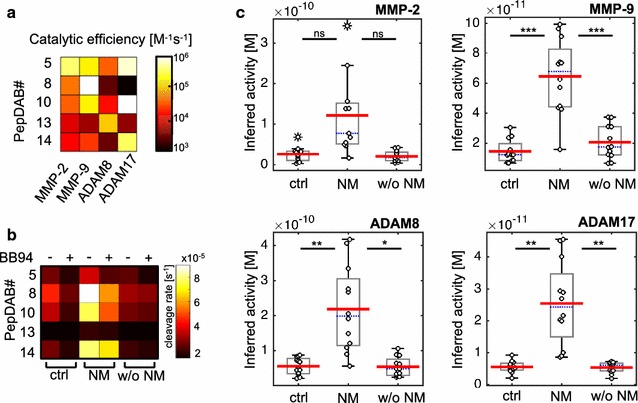
Inferred protease activitity of MMP-9, ADAM8 and ADAM17 in CSF is increased in neoplastic meningitis. Catalytic efficiencies for FRET-based peptide substrates have been determined previously across a panel of purified recombinant enzymes [13]. Observed cleavage efficiencies for MMP-2, MMP-9, ADAM8 and ADAM17 for PEPDab Substrates 5, 8, 10, 13 and 14 (modified from [13] are shown **a**. Effects of broad-spectrum MMP-inhibitor batimastat (BB-94+) treatment on observed substrate cleavage in representative CSF samples. Combination of specific substrates and inhibitors allow to infer protease activities accurately (**b**). PrAMA inferred the cleavage signatures from (Fig. 1a), using parameters from (**a**). PrAMA inference results in CSF in patients with NM were compared to control individuals and patients with brain metastases w/o NM. The *box* indicates the interquartile range, *blue bar* indicates the median, *red bar* denote the mean values and *whiskers* indicate the 95% confidence interval. *Open dots* represent the individual samples (o), extreme values are marked with *asterisks*. Inferred differences were statistically significant for MMP-9, ADAM8 and ADAM17, stars indicate * p <0.01; ** p <0.005, *** p <0.001 (**c**)

Individual time-lapse measurement derived from relatively non-specific FRET-substrates can not generally be understood to relate to specific proteases. To address this issue, PrAMA inference uses panels of FRET-substrate cleavage measurements, coupled with known catalytic efficiencies previously determined with recombinant enzymes [13], Fig. 2a), to elucidate particular enzyme activities from cleavage signatures obtained in clinical samples containing multiple proteases. PrAMA inference is strengthened by comparison of reaction rates in the presence and absence of specific inhibitors [13]. We analyzed all CSF samples using a set of five PEPDab substrates along with the broad-spectrum MP inhibitor batimastat (BB-94). Addition of 1 μM BB-94 reduced the observed rates by at least 50% on average, supporting that although MMP and ADAM activities are the major components of the observed substrate cleavage, other non-MMP/ADAM protease activities are present in the samples (Fig. 2b). PrAMA inference of specific protease activities revealed significant differences between crtl and NM for MMP-9 (4.5-fold, p = 9.9 × 10^−6^), ADAM8 (4.7-fold, p = 0.00054) and ADAM17 (4.8 fold, p = 0.00051). Changes for MMP-2 were not significant, ADAM10 was not identified in the enzyme composition of the CSF samples by PrAMA. Of the proteases identified, PrAMA inference suggests the activities of MMP-9 and ADAM17 were the highest on average (Fig. 2c). To confirm that the observed peptide cleavage patterns are correlated with NM and not with a general presence of a malignancy, we included a patient cohort in our analyses with metastases but without leptomeningeal involvement.

### Substrate cleavage patterns in CSF in follow-up lumbar CSF punctures

**Fig. 3:**
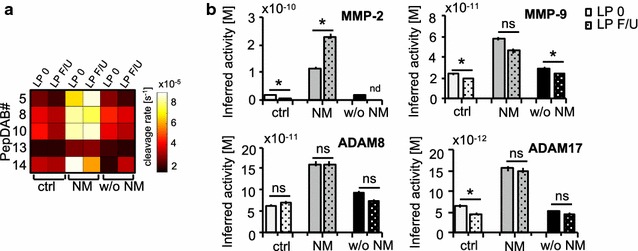
PrAMA inference results are reproducible in follow-up lumbar punctures (LP). Substrate cleavage patterns in CSF samples obtained from same patients with unchanged clinical diagnosis within a time period of 2 months, baseline substrate cleavage in CSF is denoted as LP 0, cleavage rates in CSF of 2-month follow-up (F/U) lumbar punctures as LP F/U. Substrate cleavage is shown for controls (ctrl), patients with neoplastic meningitis (NM) and patients with brain metastases w/o NM in a heatmap (**a**). PrAMA inferred the cleavage signatures from (Fig. 2a), using parameters from (**a**). Calculated protease activities are presented as bar graph (*light grey*, control individuals; *grey*, NM; *black*, w/o NM), *error bars* indicate standard deviation of three technical replicates, *dotted columns* denote 2-months follow-up lumbar punctures, respectively (**b**). Note that both, cleavage patterns and PrAMA inference results, remain similiar for controls, NM and the patient with brain metastases w/o NM. Two-tailed, one-sample *t test* was used to compare initial protease activities with 2-month follow-up measurements, values were not significant (ns, p ≥ 0.05) or significant * (p ≤ 0.05)

Following initial diagnosis of NM, patients are undergoing regular follow-up lumbar punctures in the clinical routine for intrathecal chemotherapeutic treatment and monitoring of disease progression. CSF samples obtained from patients who underwent 2-month follow-up lumbar punctures with cytologically proven stable diagnosis were subjected to PrAMA to demonstrate reproducibility of results. For all patient samples analyzed, substrate cleavage patterns in the CSF remained similar compared to baseline cleavage (Fig. 3a). Consistently, the inferred protease activities resembled baseline activities for ADAM8, ADAM17 and MMP-9 as observed in the respective CSF (Fig. 3b).

### Correlation of protease activities with CSF cell counts and BBB disturbance

Conflicting results concerning the relationship of protease expression and CSF cell count have been reported for certain MMPs, and so far, only few studies have investigated the biological activity of proteases in CSF. For MMP-9, there were studies describing either a positive or a non-linear correlation of protein levels, CSF cell count and blood–brain-barrier impairment in various neurological diseases [21, 24, 27, 40].

To overcome this problem, we sought to use the PrAMA method that allows for sensitive detection of biochemical parameters reflecting the process of cellular invasion into the leptomeningeal space. Therefore we analyzed 16 patients presenting with pleocytosis in CSF cytology, including patients with neurological pathologies other than NM, to elucidate the relationship between substrate cleavage, cell count and CSF/serum albumin ratio, a routine parameter indicating blood–brain-barrier disturbance. Pearson’s correlation statistics revealed a significant correlation of substrate cleavage rates and BBB impairment (expressed by the QAlb, albumin quotient) with a high to good correlation coefficient (r) depending on the inclusion of one patient (Additional file 1: Figs. S2 and S3). In contrast, cleavage rates and CSF cell count did not correlate, as no correlation coefficient was higher than 0.075 (Additional file 1: Fig. S3). The cellular source of the observed protease activity remains unclear; however, our results indicate that mere appearance of certain cell populations in the CSF does not reflect their biological activation. Notably, increased, uncontrolled MMP activation is implicated to play crucial roles in cerebral pathophysiology by mediating disruption of the blood–brain-barrier integrity and worsening the outcome [37, 38, 41]. In accordance, BBB disruption may be a result of increased protease activity in the CSF and vice versa.

### Therapeutic progression monitoring of neoplastic meningitis by PrAMA

**Fig. 4:**
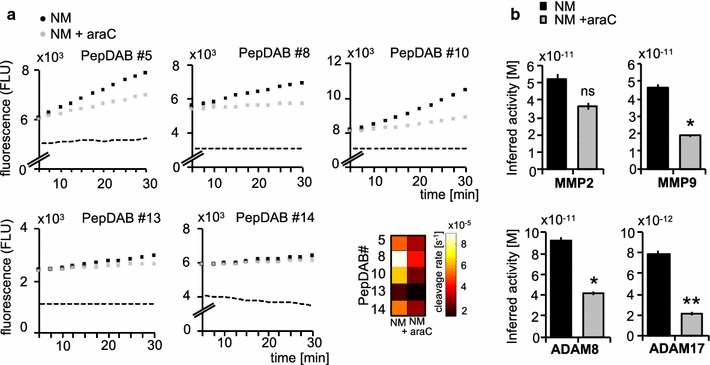
Protease activity decreases following intrathecal treatment with liposomal cytarabin. In a 62-year old breast cancer patient with leptomeningeal metastasis, protease activities were analyzed prior and after intrathecal treatment with liposomal cytarabin. Time-lapse fluorimetry output of the same patient with neoplastic meningitis arising from breast cancer previous (NM, black dots) and following treatment of NM with Cytarabin (araC) (NM + AraC, grey dots) for 3 months. Substrate tracking (PEPDab 5, 8, 10, 13 and 14) for 30 min averaged over 3 technical replicates is shown, *dashed line* indicates the negative control. Cleavage rates were calculated as described previously using a non-linear kinetic model to fit the time-lapse data (**a**). PrAMA inference results for samples analysed in A, using significance threshold σ_T_ = 1.4. *Error bars* indicate standard deviation of technical triplicates (**b**). Two-tailed, one-sample *t test* was used to compare cleavage rates pre and post treatment with araC. Values were not significant (ns, p ≥ 0.05), significant * (p ≤ 0.05) or highly significant ** (p ≤ 0.01)

The key factor in the outcome of NM is early diagnosis, because even though clinically manifest NM is treated aggressively with a combination of radiation, systemic and intrathecal chemotherapy, therapy is palliative with the goal to extend survival and improve neurologic symptoms [6]. In order to evaluate whether PrAMA can be used to monitor therapy outcome, a case of a 62-year old breast cancer patient with leptomeningeal metastasis was assessed with collection of CSF samples before treatment and 3 months after intrathecal chemotherapy with liposomal cytarabine (cytosine arabinoside, araC, Fig. 4). Following treatment with araC for 3 months, substrate cleavage rates and inferred protease activities decreased by 2.3-, 2.4.- and fourfold for ADAM8, MMP-9 and ADAM17, respectively, after intrathecal treatment with araC, whereas the MMP-2 activity was not significantly changed. Coincidently the status of the patient appeared stable over 2 years together with continuously low protease activities. Thus, protease activity profiles may have clinical implementation to sensitively monitor treatment efficacy and disease progression (Fig. 4).

## Discussion

Since abnormalities on MRI or in CSF require a substantial extent of disease in the case of leptomeningeal metastasis, it is likely that treatment delay due to the insensitivity of current methodologies contributes to the poor prognosis in NM. On the molecular level, CSF is a heterogeneous mixture of a low number of leukocytes, debris, small amounts of soluble protein, and tumor cells in the case of NM. The cellular components in the CSF are an important source of proteins determining the course of the disease relevance. Small molecules deriving from the brain or the blood reach the CSF compartment via diffusion. Conventional analytic approaches such as ELISA or Western Blot are often not suitable to detect low-abundance protein biomarkers in the CSF, which is characterized by a very low concentration of proteins. Compositional mass spectrometry-based proteomic studies of the CSF help to gain insight into dynamic disease-related changes, but generally are too complex for clinical routine [42]. Here we attempted to overcome this diagnostic constraint by providing a sensitive method of detecting increased proteolytic activities in small volumes of CSF samples only in NM patients with a reproducible and significant difference to samples from patients with brain metastasis and control patients.

MMP2, MMP-9, ADAM8, and ADAM17 are increased in CSF samples from NM patients. For a single case of a clinically stable patient, that is with no signs of impairment based on cytology, MRI and neurological examination, we observed almost similar activity values with the exception of MMP-2, which slightly increased in the follow-up sample. Moreover, in another patient treated intrathecally with the antimetabolite cytarabine, the increase of MMP-2 and all other proteases examined could be reverted, suggesting that the therapeutic progress is reflected by a decrease in MMP and ADAM activities as observed here.

Although we can clearly distinguish CSF samples from NM patients in contrast to control individuals and patients with brain metastases, the result of the PrAMA analysis has some limitations: firstly PrAMA inference suggests a major contribution of distinct proteases to the observed metalloprotease activities, as they can be partially inhibited by BB-94 that works on MMP-2, MMP-9, ADAM8, ADAM17; secondly, based on activities not inhibited by BB-94, we postulate protease activities other than metalloproteases present in CSF samples.

With respect to the identity of the metalloproteases, we have attempted to confirm their presence by ELISA. Only in a very severe case of NM, a 53-year old patient with progressive breast cancer with severe neurologic deficits and diagnosis based on CSF cytology, we were able to detect MMP-9 and TNF-RI (as ADAM17 substrate) by ELISA (Additional file 1: Fig. S4). From this CSF sample, activities determined by PrAMA were up to 10-times higher than in all other NM cases. According to Dixon’s Q-Test, this sample was defined as outlier and excluded from cluster analysis. Clusters of atypical, malignant cells dominated the cytogram and the blood–brain-barrier function was strongly impaired as determined by an albumin quotient of 80.4. Although multimodal treatment was initiated immediately, NM in this patient progressed to death within 3 months.

Although our results provide pilot data for the high impact of CSF profiling in disease at present, they underscore the value of multiplexing, i.e. with respect to a microfluidic platform [43] and to a series of other diseases related to CSF changes. Future studies in larger patient cohorts are required to validate our preliminary results. Mechanistically, invasion of malignant cells into the leptomeninges and the CSF is a multistep process that requires differential expression of migration- and invasion-related proteins. On a time scale, disease-related changes in the composition of the CSF are expected to precede transmigration of cells into the CSF. Most likely, the proteolytic activity in the CSF may increase before pathological signs can be detected by MRI or CSF cytology. The case of a 62 year-old patient with minimal CSF involvement from breast cancer surviving more than two years, which is described before, underlines the clinical experience that early detection and subsequent treatment of malignant cells in the CSF is crucial for long-term control of neoplastic meningitis. Very early detection of subclinical CSF invasion may therefore be crucial to improve the otherwise frustrating results of NM treatment.

Clinical data known to date implicate that conventional therapies are not sufficient to achieve long-term control or remission in cases of neoplastic meningitis (Strik, Proemmel 2010). Molecularly targeted therapies that aim at preventing tumor cells from invasion into the CSF space may offer more efficacy. At present, several pharmacological inhibitors of MPs are already available and ADAM-inhibitors are being developed. After a more detailed analysis of the pathophysiological mechanisms, pharmacological inhibition of MPs and ADAM proteins may be efficient tools to prevent or treat malignant CSF-invasion.

In this respect, our results from a multiplexed assay provide a novel perspective into MMP/ADAM contribution to the pathophysiology of NM. More importantly, the simple profiling of protease activities in CSF samples from patients with medical indications seems to reflect individual context-dependent protease function in invasive diseases and should be explored in a larger cohort of NM patients but also in other clinical applications including infectious diseases in the CNS.

## Conclusion

Based on these pilot data we demonstrated that the simultaneous detection of metalloprotease activities in CSF samples by the PrAMA method could be of diagnostic value to distinguish NM from metastasis without NM and from controls with normal CSF. The observed cleavage profiles are consistently present in patients with a positive follow-up analysis and are modulated by successful treatment of NM. Moreover, we detected ADAM8, MMP-9, and ADAM17 activities in the CSF as major analytes in the CSF of patients with NM.

## Additional file

**Additional file 1.** Sketch of proteases and CSF infiltration, correlation analyses, and ELISA results.

## Abbreviations

ADAM: A Disintegrin And Metalloprotease
araC: cytarabin
BB-94: batimastat
BBB: blood-brain-barrier
Crtl: control
CSF: cerebrospinal fluid
ECM: extracellular matrix
ELISA: enzyme-linked immunosorbent assay
FRET: fluorescence resonance energy transfer
MMP: matrix-metallo-Protease
MP: metallo-protease
nd: not detectable
NM: neoplastic meningitis
ns: not significant
PrAMA: proteolytic activity matrix assay
QAlb: albumin quotient
w/o: without
