# Supplementary material for: Profiling of metalloprotease activities in cerebrospinal fluids of patients with neoplastic meningitis
Source: Fluids Barriers CNS. 2017 Aug 14;14:22. doi: 10.1186/s12987-017-0070-5 (PMC5556623; doi:10.1186/s12987-017-0070-5)
Supplement: Supplementary file 1 — Additional file 1. Sketch of proteases and CSF infiltration, correlation analyses, and ELISA results. [file 12987_2017_70_MOESM1_ESM.pdf]

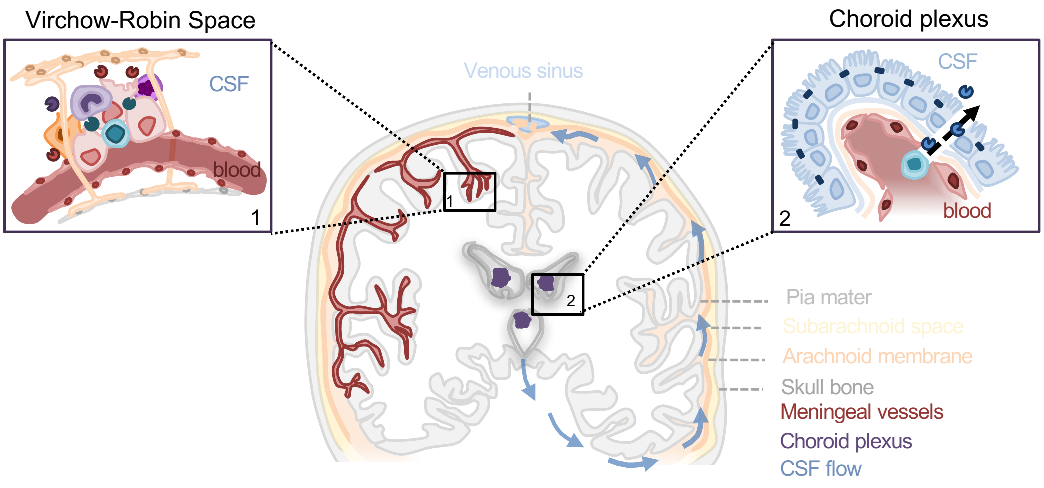

CSF (cellular composition)		
Physiological	Meningeosis	Meningitis
Lymphocytes (few)<br>• T-Cells (80%)<br>• Monocytes (5%)<br>• B-Cells (1%)	Tumor cells<br>Lymphocytes ⇧<br>Monocytes ⇧	Neutrophils ⇧<br>(bacterial)<br><br>Lymphocytes ⇧<br>(viral)

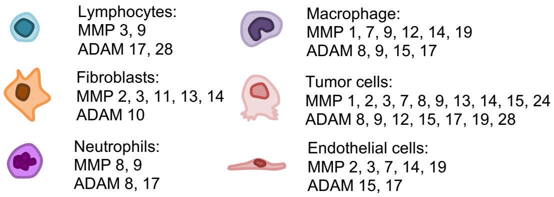

Supplemental Figure S1: Dissemination of cells and origin of extracellular proteases in the CSF. The cerebrospinal fluid (CSF) is actively secreted by the epithelium of the choroid plexus (violet) located in the ventricles of the brain. The CSF circulates from the ventricles to the subarachnoid space and is resorbed into the venous sinuses through the arachnoid villi (CSF flow; light blue). Lymphocytes transmigrate across the fenestered endothelium and the tight epithelium of the plexus choroideus into the CSF modulated by adhesion ligands and promoted by metalloproteases such as MMP-3 and MMP-9, as well as ADAM17 and ADAM28 (2). In contrast, circulating tumor cells follow the hematogenic route, get trapped in the meningeal vessels and colonize in the leptomeninges. MMPs and ADAMs secreted by tumor cells mediate important steps in metastatic seeding and dissemination into the CSF. Tumor-associated stromal and immune cells associate with tumor cells and release specific MMPs and ADAMs (1). In pathologic states in the CNS the cellular composition of the CSF changes which affects the protease profiles (table, bottom left). The relevant MMPs and ADAMs that stem from respective cell types are indicated (bottom right).

Supplemental Figure S1

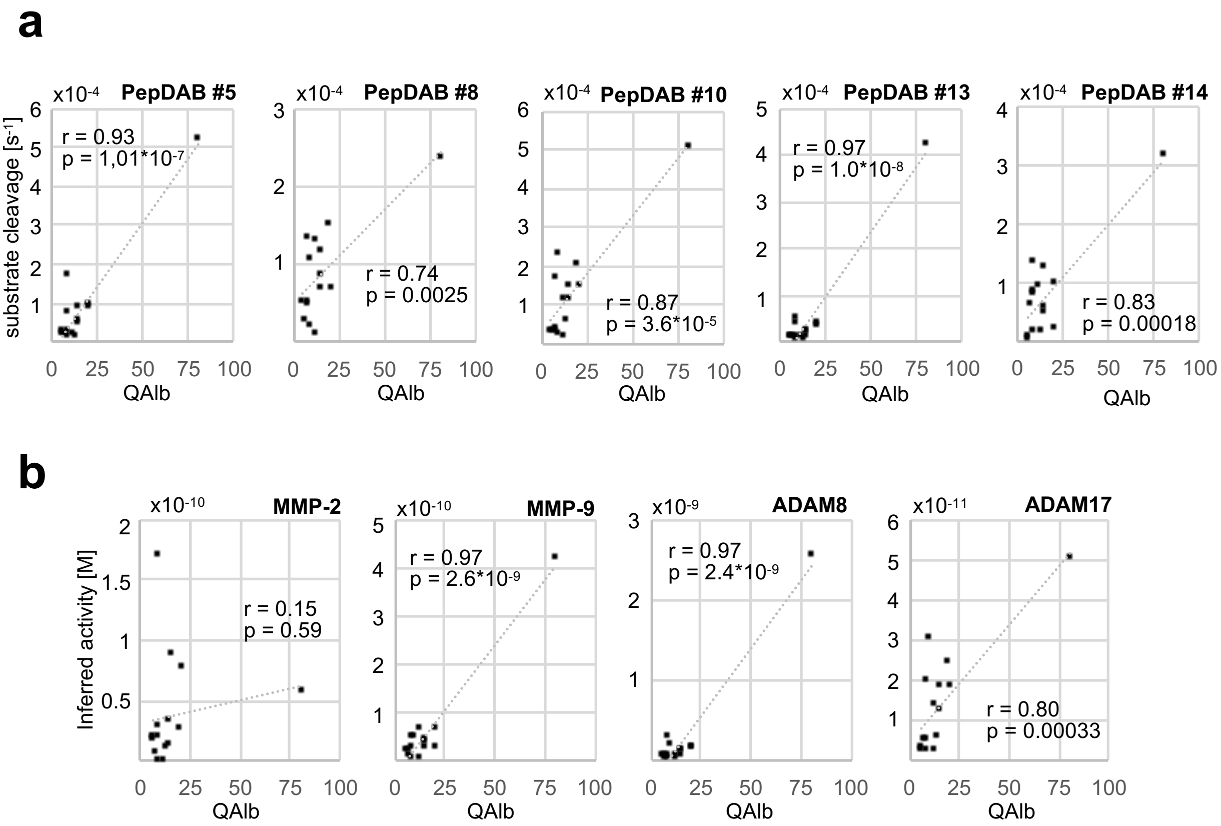

Figure S2: Correlation analysis for blood-brain-barrier impairement and protease activity. To evaluate the relationship between blood-brain-barrier impairement (QAlb), cleavage rates of PepDAB substrates and inferred protease activity, Pearson‘s correlation coefficient was calculated, respectively. (A) Scatter plots and corresponding regression line represent the positive correlation between substrate cleavage for each peptide substrate (PepDAB #5, PepDAB#8, PepDAB #10, PepDAB#13 and PepDAB#14). Pearson‘s correlation coefficient (r) with p-value (p) is indicated in each graph. (B) Correlation analysis for inferred protease activities revealed significant positive correlation for MMP-9 ($r=0.97$, $p=2.6*10^{-9}$), ADAM8 ($r=0.97$, $p=2.4*10^{-9}$) and ADAM17 ($r=0.80$, $p=0.00033$), whereas there was no relationship between blood-brain-barrier impairement and inferred MMP-2 activity.

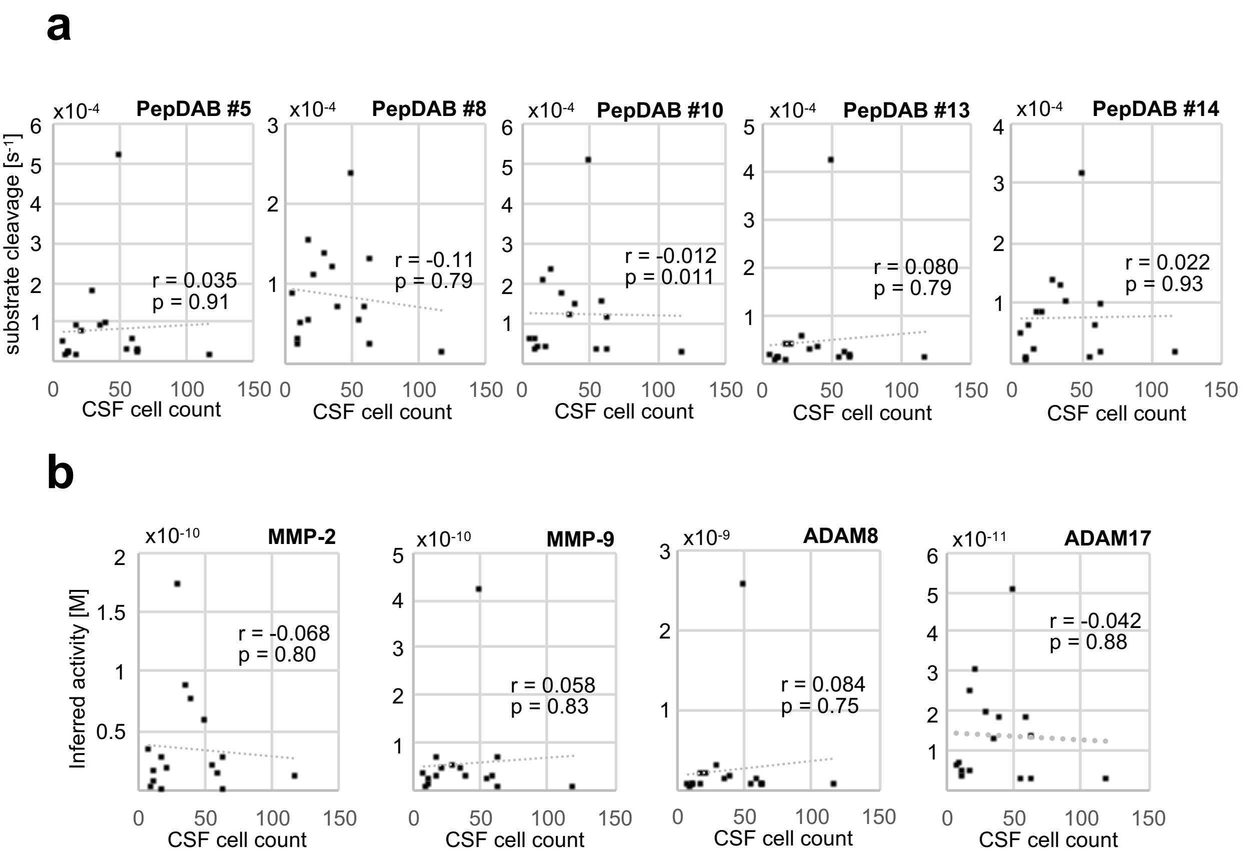

Figure S3: Correlation analysis for CSF cell counts and protease activities. To evaluate the relationship between CSF cell count, cleavage rates of PepDAB substrates and inferred protease activity, Pearson‘s correlation coefficient was calculated, respectively. (A) Scatter plot and corresponding regression line for substrate cleavage against CSF cell count reveals no significant correlation. (B) Accordingly, correlation analysis of inferred activities (MMP-2, MMP-9, ADAM8 and ADAM17) was not significant. Pearson‘s correlation coefficient (r) with p-value (p) is indicated in each individual graph.

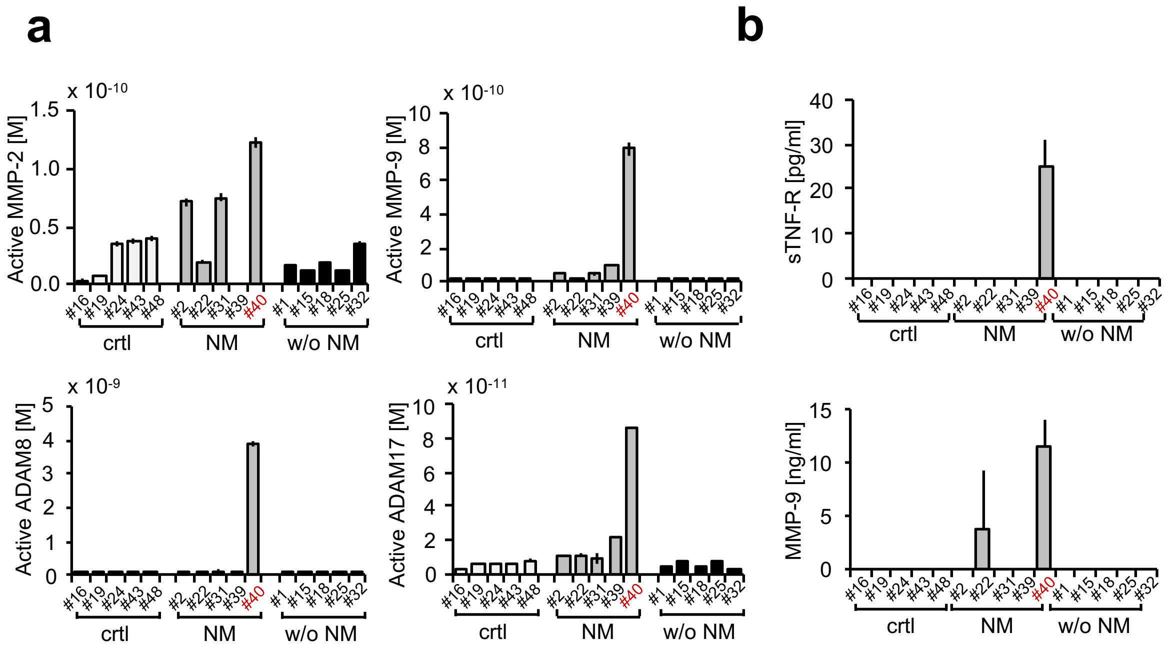

Figure S4: Inferred protease activites (A) and ELISA results (B) in a subset of ctrl, NM and w/o NM CSF samples. Cell-free CSF samples containing sufficient volumes were subjected to both, PrAMA (A) and ELISA (B) for soluble TNF-receptor I (sTNF-RI), and MMP-9. Cleavage rates for all substrates and inferred protease activities were highest for NM #40 (red label). According to Dixon‘s Q-Test, this sample was defined as outlier and excluded from cluster analysis. However, this particular sample showed the highest protease activities which were in average 8-10-times higher than in all other CSF samples. The corresponding ELISA results for soluble TNF-RI (sTNF-RI, a ADAM17 substrate) and MMP9 were slighty above the detection limit for the ELISA analyses, so that in all other CSF samples, even when concentrated, ADAM17 and MMP9 were not detectable. ELISA analyses were performed according to the manufacturer‘s instructions with a limit of detection of 12.5 pg/ml for sTNF-RI and 7.8 ng/ml for MMP-9.

Supplemental Figure S4

PepDAB#	Substrate Sequence
5	Dabcyl-Leu-Ala-Gln-Ala-Homophenylalanine-Arg-Ser-Lys(5-FAM)-NH
8	Dabcyl-Pro-Cha-Gly-Cys(Me)His-Ala-Lys(5-FAM)-NH
10	Dabcyl-Ser-Pro-Leu-Ala-Gln-Ala-Val-Arg-Ser-Ser-Lys(5-FAM)-NH2
13	Dabcyl-His-Gly-Asp-Gln-Met-Ala-Gln-Lys-Ser-Lys(5-FAM)-NH2
14	Dabcyl-Glu-His-Ala-Asp-Leu-Leu-Ala-Val-Val-Ala-Lys(5-FAM)-NH2

Table S1: Synthetic polypeptide FRET-substrates and their amino acid composition. Reference numbers denote substrate indices used throughout the manuscript.
